# Supplementary material for: Enhancing DLG2 Implications in Neuropsychiatric Disorders: Analysis of a Cohort of Eight Patients with 11q14.1 Imbalances
Source: Genes (Basel). 2022 May 12;13(5):859. doi: 10.3390/genes13050859 (PMC9140951; doi:10.3390/genes13050859)

Table S1: Anamnesis, exams and physical features of the patients

Pt	1	2	3	4	5	6	7	8
Pre-perinatal parameters and information	Spontaneous vaginal birth at term<br>W: 2,910 g<br>L: NA<br>OFC: NA<br>Apgar scores: 10 (5')	Spontaneous vaginal birth at term<br>W: 2,990 g<br>L: 49 cm<br>OFC: NA<br>Apgar scores: NA	Spontaneous vaginal birth at term<br>W: 3,100 g<br>L: 49 cm<br>OFC: NA<br>Apgar scores: NA	Alcohol assumption during pregnancy<br>Born by cesarean section at 41st gw.<br>W: 2800 g (SGA)<br>L 47 cm. OFC: NA<br>Apgar scores: NA<br>Perinatal sufferance, neonatal sepsis, diaphragmatic lymphoadenopathy, feeding difficulties	Spontaneous vaginal birth at term<br>W: 2,750 g (SGA)<br>L: 49 cm<br>OFC: 35 cm<br>Apgar scores: 9 (1'), 9 (5')	Born by planned cesarean section at term<br>W: 2,600 g (SGA)<br>L: NA<br>OFC: NA.<br>Apgar scores: NA	Born at 31st GW by cesarean section due to maternal gestosis and oligohydramnios<br>W: 1100 g (SGA)<br>L: 34 cm<br>OFC: 27 cm<br>Apgar scores: 5 (1'), 5 (5')	Spontaneous vaginal birth at term (41st GW + 5 days)<br>by spontaneous delivery.<br>W: 3550 g<br>L: 58 cm<br>CC: 38 cm<br>Apgar scores 10, (1'), 10 (5')
Family history	Negative	NA	Negative	Learning difficulties and attention deficit reported in his father	Motor dyspraxia, congenital clubfoot (Father)<br>ASD (maternal cousin)	Epilepsy and depressive mood in maternal line; ASD and ID in his sister (negative for 11q14 deletion).	ASD, anxiety and depressive mood in maternal line	LD in paternal line.<br>Social difficulties and academical interests in maternal line.
Instrumental and other genetic exams	Neurometabolic workup: N<br>ECG: N	*FMR1* analysis: N	Ophtalmologic evaluation: N<br>Audiologic evaluation: N<br>ECG: N<br>FMR1 analysis: N	Ecocardiography: N<br>Abdominal ultrasound: N<br>PTPN11 analysis: N<br>SOS1 analysis: N<br>SHOX analysis: N	ABR: Acoustic threshold 50 dB nHL in right ear and 20 dB nHL in left ear<br>Dermatologic evaluation: Three cutaneous angiomas<br>Thyroid function evaluation and celiac screening: N	ECG: N<br>Echocardiography: N<br>Hormone dosage: mild ACTH, TSH and insulin increase<br>FMR1 analysis: N	Echocardiography: interatrial septum defect and patent foramen ovale.<br>ECG: N<br>FMR1 analysis: N	ECG: N<br>Abdominal ultrasound: N<br>FMR1 analysis: N<br>Metabolic workup: gluten intolerance
Physical examination	No dysmorphic features	Microcephaly, up-turned palpebral rims, large incisors, micrognathy, simple auricles, sparse eyebrows in the distal-third, clinodactyly of the fifth finger, big hands' and feet's thumbs, hyperchromic mark on the right cheek and on the left thigh	Frontal bossing, high hairline, down-turned palpebral rims, pointed chin	Slow growth<br>GH deficit	Flat angioma on the right parietal area of the head, on the right region of the chest and on the left palm	Obesity<br>Acantosis nigricans<br>Rubrae Striae<br>Elongated eyelid rims<br>Tapered Fingers	Hypospadia<br>Tapered fingers<br>Bilateral Sandal Gap<br>Overweight	Macrocephaly

ABR: Auditory Brainstem Responses; ASD: Autism Spectrum Disorder; ECG: electrocardiogram; GH: Growth hormone; GW: gestational week; ID: Intellectual Disability; IUGR: intrauterine growth retardation; L: length; LD: Language disorder; N: Normal; NA: not available; OFC: occipito-frontal circumference; Pt: patient; SGA: Small for gestational age; W: Weight.

Table S2: Genes harbored in the additional CNVs detected in patient 1, 3 and 6.

GENE	NAME	FUNCTION	ESPRESSION	DISEASE
**Patient 1**				
TMEM126B	Transmenbrane protein 126B	This gene encodes a mitochondrial transmembrane protein which is a component of the mitochondrial complex I assembly complex. The encoded protein serves as an assembly factor that is required for formation of the membrane arm of the complex.	Ubiquitous	Mitochondrial complex I deficiency, nuclear type 29 (#618250)
TMEM126A	Transmenbrane protein 126A	The protein encoded by this gene is a mitochondrial membrane protein of unknown function. Defects in this gene are a cause of optic atrophy type 7 (OPA7).	Ubiquitous	Optic atrophy 7 (#612989)
CREBZF	CREB/ATF bZIP transcription factor	Involved in negative regulation of gene expression, epigenetic; regulation of transcription, DNA-templated; and response to virus.	Ubiquitous	
CCDC89	Coiled-coil domain containing 89	Unknown. Predicted to be located in cytoplasm and nucleus.		
SYTL2	Synaptotagmin like 2	This protein plays a role in vesicle trafficking and controls melanosome distribution in the cell periphery.	Broad	
CCDC83	Coiled-coil domain containing 83		Testis	
PICALM	Phosphatidylinositol binding clathrin assembly protein	PICALM is involved in cellular trafficking, regulation of endocytosis, and clathrin-mediated vesicle formation. It is tightly associated with iron homeostasis and cell proliferation, and these processes are strongly tied to embryonic development	Ubiquitous	
**Patient 3**				
SLC35F3	Solute carrier family 35 member F3	Involved in thiamine transport. Predicted to be integral component of membrane	Mainly espressed in brain (RPKM 3.2), adrenal (RPKM 0.5)	
ARSB	Arylsulfatase B	Arylsulfatase B encoded by this gene belongs to the sulfatase family. The protein is targeted to the lysozyme.	Ubiquitous	Mucopolysaccharidosis type VI<br><br>(Maroteaux-Lamy) (#253200)
DMGDH	**Dimethylglycine Dehydrogenase**	Dimethylglycine dehydrogenase is an enzyme involved in the catabolism of choline, catalyzing the oxidative demethylation of dimethylglycine (DMG) to form sarcosine.	Kidney, Liver, Fat	Dimethylglycine dehydrogenase deficiency (#605850)
BHMT2	Betaine--homocysteine S-methyltransferase 2	BHMT2 is a zinc metalloenzyme that uses S-methylmethionine (SMM) as a methyl donor for the methylation of homocysteine	Kidney, Liver	
JMY	Junction mediating and regulatory protein, p53 cofactor	Predicted to be involved in several processes. MY is a stress-responsive protein involved in regulation of p53 activity. JMY also has actin-nucleating activity and plays a role in cell motility	Ubiquitous	

**Patient 6**				
VCX	Homo sapiens variable charge X-linked (VCX), transcript variant 2, mRNA	This gene belongs to the VCX/Y gene family, which has multiple members on both X and Y chromosomes, and all are expressed exclusively in male germ cells. VCX/Y genes encode small and highly charged proteins of unknown function. The presence of a putative bipartite nuclear localization signal suggests that VCX/Y members are nuclear proteins. [provided by RefSeq, Jul 2008].	Testis	
PNPLA4	Patatin/like Phospholipase Domain/Containing Protein 4	The PNPLA4 gene encodes a protein with both triacylglycerol lipase and transacetylase activities	Gene was expressed in all human tissues examined, including heart, brain, placenta, lung, liver, muscle, kidney, pancreas, and spleen	
MIR561	Homo sapiens microRNA 561 (MIR561), microRNA.			

Name, function, expression profile and the associated disease are reported for each gene

Table S3: Coding sequences involved in the CNVs of DLG2 gene.

	Exons	ENSEMBL	Genomic Coordinates	Lenght	N. aa	Aminoacidic sequence
**A**	ex 2 DLG2-220	ENSE00001469378	chr11: 85,111,735-85,111,661	75 bp	25	QNQGRCPAQNCSVEAPAWMPVHHCT
	ex 6 DLG2-203					
	ex 6 DLG2-232					
**B**	ex1 DLG2-207	ENSE00002179742	chr11:84,923,589-84,923,077	513 pb	14	**5'UTR** + MFFACYCALRTNVK
	ex1 DLG2-218	ENSE00002149728	chr11:84,923,180-84,923,077	104 pb		
	ex1 DLG2-230	ENSE00002188311	chr11:84,923,421-84,923,077	345 pb		
**C**	ex1 DLG-231	ENSE00003840932	chr11:84.720.915-84.720.296	620 pb	34	**5'UTR**+MSPVVKDPDCFTPMICHCKVACTNNTLSLMFGCK
**D**	ex7 DLG2-203	ENSE00003487678	chr11:84,534,731-84,534,570	162 pb	54	KYRYQDEDAPHDHSLPRLTHEVRGPELVHVSEKNLSQIENVHGYVLQSHISPLK
	ex 7 DLG2-232					
	ex2 DLG2-207					
	ex2 DLG2-218					
	ex2 DLG2-230					
	ex2 DLG2-231					
	ex3 DLG2-220	ENSE00002174027	chr11: 84,534,731-84,534,613	119pb	39	KYRYQDEDAPHDHSLPRLTHEVRGPELVHVSEKNLSQIE + **3'UTR**
**E**	ex1 DLG2-221	ENSE00002152856	chr11: 84,437,809-84,437,388	422pb	26	**5'UTR** + MFASIWYAKKLGRRFVHNARKAKSEK
**F**	ex1 DLG2-201	ENSE00001532570	chr11: 84,317,339-84,316,825	515pb	107	MNAYLTKQHSCSRGSDGMDAVRSAPTLIRDAHCACGWQRNCQGLGYSSQTMPSSGP GGPASNRTGGSSFNRTLWDSVRKSPHKTSTKGKGTCGEHCTCPHGWFSPAQ
	ex1 DLG2-205					
**G**	ex1 DLG2-202	ENSE00003587836	chr11: 84,273,188-84,273,151	38pb	13	**5'UTR** + XQCEQAMQHAFIP
	ex1 DLG2-229	ENSE00001469384	chr1: 84,273,280-84,273,151	130pb	35	**5' UTR** + MQRPSVSRAENYQLLWDTIASLKQCEQAMQHAFIP
**H**	Ex8 DLG2-203	ENSE00003471737	chr11:84,251,291-84,251,238	54pb	18	ASPAPIIVNTDTLDTIPY
	ex 8 DLG2-232					
	EX3 DLG2-218					
	EX3 DLG2-230					
	EX3 DLG2-207					
	EX3 DLG2-231					
	EX2 DLG2-221					
	EX2 DLG2-201					
	EX2 DLG2-205					
	EX2 DLG2-202					
	EX2 DLG2-229					

Description of the coding sequences involved in the CNVs of DLG2 gene. For each coding sequence (A to H) the corresponding exons of each transcript are reported, along with the Ensembl number, the hg38 coordinates (reverse strand), the extension in kb, the number of aminoacid encoded (N. aa), and the corresponding aminoacid sequence.

Table S4: DLG2 protein coding transcripts involved in patients' imbalances.

Trascript ID	Name	aa	Extension	Exons	Protein length (aa)	Expression information source
ENST00000376104 (mane)	DLG2-203	975aa	11: 83,455,173-85,627,344	Exons: 28<br>Coding exons 26	975	GTEx, UCSC
ENST00000650630	DLG2-232	1012aa	11: 83,457,919-85,627,270	Exons:28<br>Coding exons 27	1012	none
ENST00000472545	DLG2-215	52aa	11: 85,469,338-85,627,922	Exons: 4,<br>Coding exons: 3	52	GTEx, UCSC
ENST00000527088	DLG2-220	74aa	11: 84,534,613-85,133,123	Exons: 3,<br>Coding exons: 3	74	GTEx, UCSC
ENST00000524982	DLG2-218	866aa	11: 83,459,609-84,923,180	Exons: 24,<br>Coding exons: 24	866	GTEx, UCSC
ENST00000532653	DLG2-230	852aa	11: 83,459,789-84,923,421	Exons: 23,<br>Coding exons: 23	852	GTEx, UCSC
ENST00000398309	DLG2-207	870aa	11: 83,455,012-84,923,589	Exons: 23,<br>Coding exons: 23	870	GTEx, UCSC
ENST00000648622	DLG2-231	890aa	11: 83,457,919-84,720,915	Exons: 23,<br>Coding exons: 23	890	[9]
ENST00000527466	DLG2-221	78aa	11: 84,098,995-84,437,809	Exons: 4,<br>Coding exons: 4	78	GTEx, UCSC<br>[9]
ENST00000280241	DLG2-201	909aa	11: 83,455,012-84,317,339	Exons: 22,<br>Coding exons: 22	909	GTEx, UCSC
ENST00000398301	DLG2-205	552aa	11: 83,786,441-84,317,339	Exons: 12,<br>Coding exons: 12	552	GTEx, UCSC
ENST000003300141	DLG2-202	797aa	11: 83,459,422-84,273,188	Exons: 22,<br>Coding exons: 22	797	GTEx, UCSC
ENST00000531015	DLG2-229	811aa	11: 83,461,542-84,273,280	Exons: 21,<br>Coding exons: 21	811	GTEx, UCSC

DLG2 protein coding transcripts involved in patients' imbalances. For each transcript, the Ensembl ID is reported along with the name, the genomic coordinates (hg38) (reverse strand), the number of exons (total and coding), the protein length (aa= aminoacid), and where expression data can be found.

Figure S1: Expression of some DLG2 transcripts across tissues from GTEx Pro

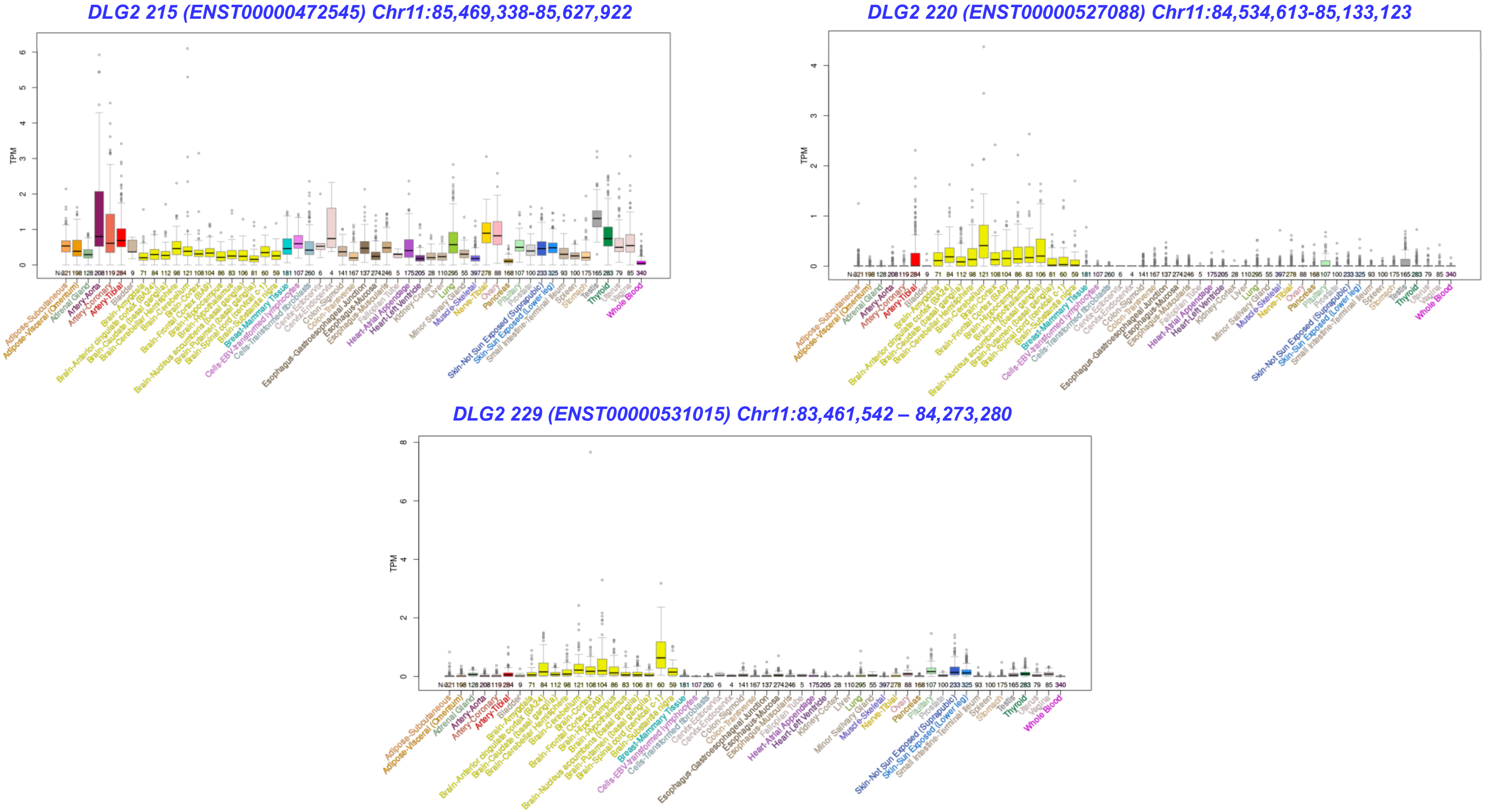

DLG2 218 (ENST00000528942) Chr11:83,459,609 – 84,923,180

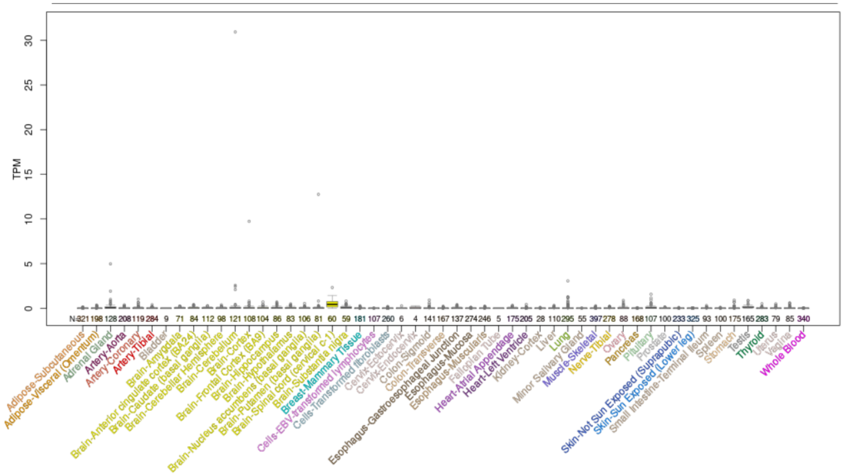

DLG2 202 (ENST00000330014) Chr11:84,459422 – 84,273,188

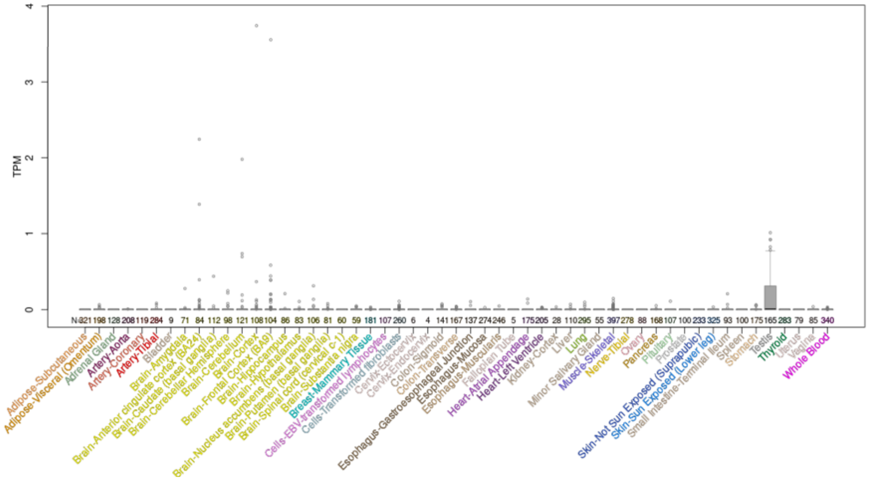

DLG2 205 (ENST00000398301) Chr11:83,786,441 – 84,317,339

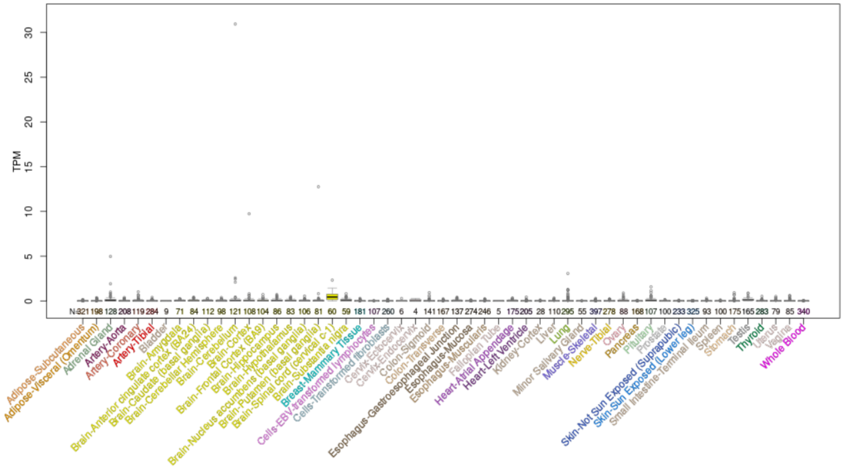

DLG2 230 (ENST00000532653) Chr11:83,459,789 – 84,923,421

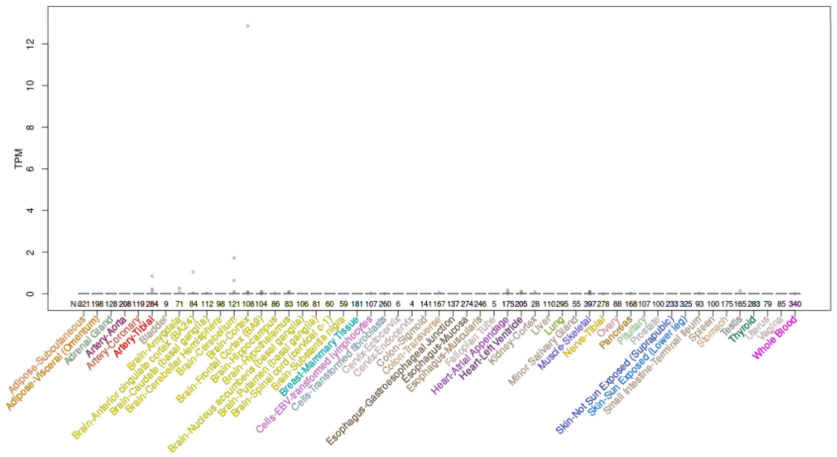

Supplement: Supplementary file 1 [file genes-13-00859-s001.zip › genes-1707395-supplementary.pdf]
